# A New Protocol of Computer-Assisted Image Analysis Highlights the Presence of Hemocytes in the Regenerating Cephalic Tentacles of Adult *Pomacea canaliculata*

**DOI:** 10.3390/ijms22095023

**Published:** 2021-05-09

**Authors:** Giulia Bergamini, Mohamad Ahmad, Marina Cocchi, Davide Malagoli

**Affiliations:** 1 Department of Life Sciences, University of Modena and Reggio Emilia, 41125 Modena, Italy; giulia.bergamini@unimore.it; 2 Department of Chemical and Geological Sciences, University of Modena and Reggio Emilia, 41125 Modena, Italy; mohamad.ahmad@unimore.it (M.A.); marina.cocchi@unimore.it (M.C.); 3 Laboratoire de Spectroscopie pour les Interactions, la Réactivité et l’Environnement, LASIRE, CNRS, UMR 8516, University of Lille, Villeneuve-d’Ascq, 59650 Lille, France

**Keywords:** adult regeneration, apple snail, blastema, hemocyte, gastropod, image analysis, immunity, mollusk

## Abstract

In humans, injuries and diseases can result in irreversible tissue or organ loss. This well-known fact has prompted several basic studies on organisms capable of adult regeneration, such as amphibians, bony fish, and invertebrates. These studies have provided important biological information and helped to develop regenerative medicine therapies, but important gaps concerning the regulation of tissue and organ regeneration remain to be elucidated. To this aim, new models for studying regenerative biology could prove helpful. Here, the description of the cephalic tentacle regeneration in the adult of the freshwater snail *Pomacea canaliculata* is presented. In this invasive mollusk, the whole tentacle is reconstructed within 3 months. Regenerating epithelial, connective, muscular and neural components are already recognizable 72 h post-amputation (hpa). Only in the early phases of regeneration, several hemocytes are retrieved in the forming blastema. In view of quantifying the hemocytes retrieved in regenerating organs, granular hemocytes present in the tentacle blastema at 12 hpa were counted, with a new and specific computer-assisted image analysis protocol. Since it can be applied in absence of specific cell markers and after a common hematoxylin-eosin staining, this protocol could prove helpful to evidence and count the hemocytes interspersed among regenerating tissues, helping to unveil the role of immune-related cells in sensory organ regeneration.

## 1. Introduction

Invertebrates have provided important contributions to the understanding of human biology, and their usage in biological research has been increasing in the last few decades [1,2,3]. Among the biological issues addressed by comparative research, regeneration and its relationship with the immune system has attracted attention for a long time [1,4]. Many invertebrate models present relevant regeneration capabilities [2,5,6] while in Amniotes, this capability is significantly reduced [7] and the adaptive immune system is frequently considered as detrimental for a complete regeneration [8]. The innate immune components of invertebrates such as in annelids [9,10] and molluscs [11,12,13] seem to play a major role in regulating the regeneration process. Among gastropods, information about the regeneration of neural components derives from experiments and observations on land snails and slugs [14,15], and from studies on aquatic species. Among aquatic gastropods, studies in the freshwater snail *Lymnaea stagnalis* demonstrated the crucial role played by resident phagocytes for the regeneration of neural components, a feature shared by several invertebrate species, suggesting an ancient origin and conserved relationships between phagocytic cell functions and neural regeneration [16]. Scanty information about regeneration is available for the freshwater snail, *Pomacea canaliculata*, a highly invasive snail [17]. Adult *P. canaliculata* can regenerate the shell [18], the camera-type eye [19,20,21], and the oral and cephalic tentacles [22] but, to our knowledge, no information is available about the relationship between the regeneration process in sensory organs and innate immune components.

Cephalic tentacles play a major role in *P. canaliculata* biology, as they are utilized for food search, co-specific recognition, and orienting [23,24]. These long tentacles present a well-defined organization, with muscular and connective tissue representing a sort of scaffold for the nervous component that serves the olfactory epithelium [23]. In order to provide novel information on the regeneration ability of *P. canaliculata*, and to correlate regeneration with the presence of immune-related cells, the cephalic tentacle regeneration in adult *P. canaliculata* was documented. In parallel, we wanted to investigate the presence of the immune-related cells, i.e., hemocytes, in the regenerating tentacle. Circulating hemocytes of *P. canaliculata* have been classified, by flow cytometry and optical microscopy into two morphological categories, namely Group I and Group II hemocytes [25]. Group I hemocytes include small cells, with a central and round nucleus. Their morphology recalls that of blast-like cells and they do not seem able to perform phagocytosis on heat-inactivated bacteria. Group I hemocytes also includes hemocytes with intermediate morphology between Group I and Group II hemocytes. Group II hemocytes represent the majority of circulating hemocytes. They are larger and can present either agranular or granular cytoplasm. They phagocytize heat-inactivated bacteria. Thanks to the stainability of their cytoplasmic granules, granular hemocytes present a distinct morphology [25].

Image analysis has been applied to invertebrate hemocyte studies since the end of the last century [26,27]. The increasing availability of molecular data and cell markers opened the possibility for advanced image analysis, both through flow cytometry and optical microscopy. The most advanced methods of image analysis can identify a variety of cell types in the context of healthy and pathological tissues relying on the availability of multiple cell markers [28]. In the absence of specific cell markers, advanced flow cytometry can help [29], but it does not provide information about tissue contextualization [28]. To our knowledge, no specific molecular markers are available for tracking tissue and circulating hemocytes in *P. canaliculata* [25,30,31], hampering hemocyte quantification outside the hemolymph. On these basis, histological techniques and a new protocol of computer-assisted image analysis were combined to document and count the hemocytes in the regenerating cephalic tentacles. The developed image analysis method can automatically identify hemocytes within hematoxylin-eosin stained tissues. The proposed method shares some similarity with approaches currently used for cell shape identification and counting [32], with a significant point of novelty in the identification phase preceding the phase of hemocyte counting. Differently from current methodologies, our identification phase is based on multivariate image analysis to build a reference model of hemocyte textural/color features, benefiting from the simultaneous analysis of pixel-neighbours at all color channels. Moreover, using a Principal Component Analysis (PCA) model, our method can automatically evaluate images acquired with the same microscope configuration without operator intervention. Our morphological observations and software-based quantifications indicate that in the immediacy of amputation, Group II granular hemocytes increase significantly in the forming blastema.

## 2. Results

### 2.1. Histological Description of P. canaliculata Cephalic Tentacle Anatomy

**Figure 1:**
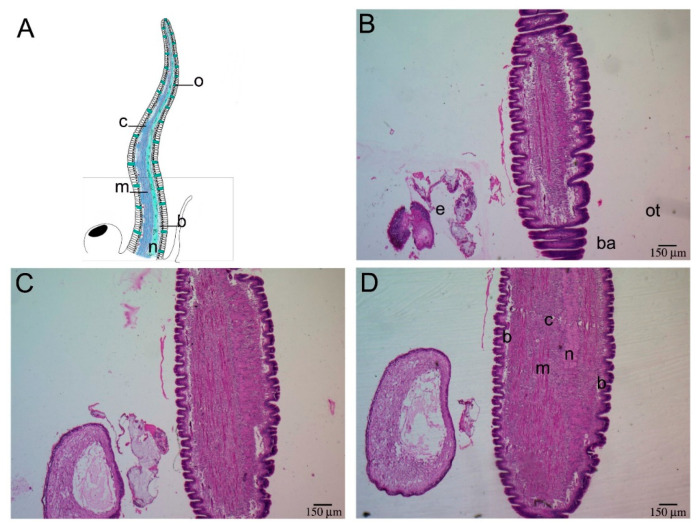

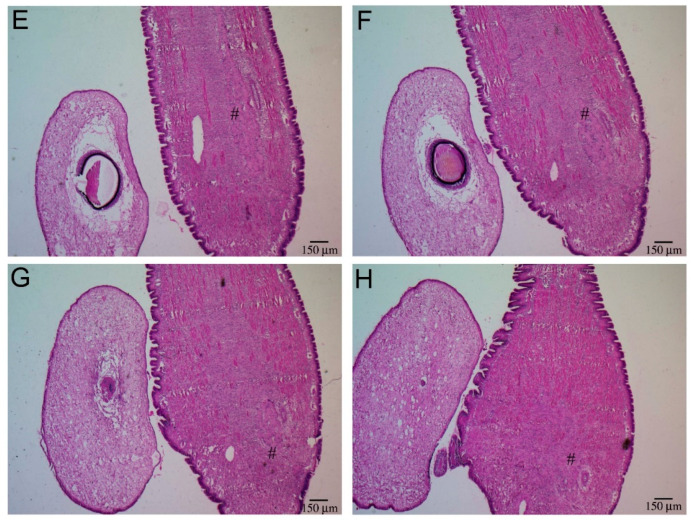
Base-to-tip histological analysis of *P. canaliculata* model cephalic tentacle. Longitudinal sections of the tentacle base. (**A**) Illustration summarizing cephalic tentacle components; the tentacle nerve (n) was situated close to the oral side, whereas connective (c) and muscular (m) tissues were more evenly distributed than represented in the illustration. The olfactory cells (o) run along the two sides of the tentacle. The rectangle relates to the portion pictured in the histological micrographs; (**B**–**H**) Longitudinal sections of the base of the cephalic tentacle, taken from the dorsal to the ventral face, representing the whole tentacle thickness. (**B**) At the first section presented, the eye (e) on the left was already visible. The position of oral tentacle (ot) and basal origin of the tentacle (ba) have been indicated for helping orientation; (**C**,**D**) Moving along the dorsal–ventral axis, the histological micrographs confirmed the presence of all the components; (**E**–**H**) the structure distributed more asymmetrically was the tentacle nerve, that at the base of the tentacle seemed to emerge from an enlarged neural component (**#**), that remained as the only neural structure visible on the extreme ventral portion of the tentacle base. Abbreviations: b, blood-containing lacunae; ba, basal origin of the tentacle; c, connective tissue; e, eye; m, muscle fibers; n, tentacle nerve; o, olfactory cells [24]; ot, oral tentacle.

**Figure 2:**
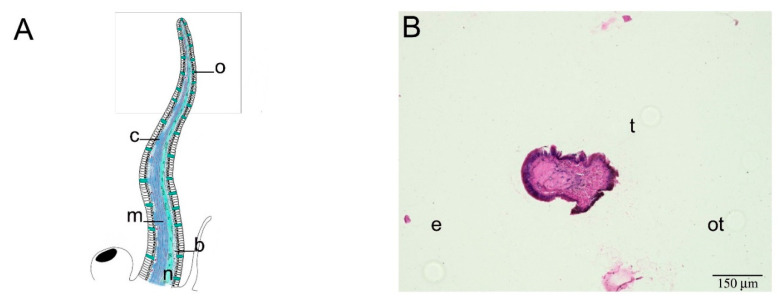

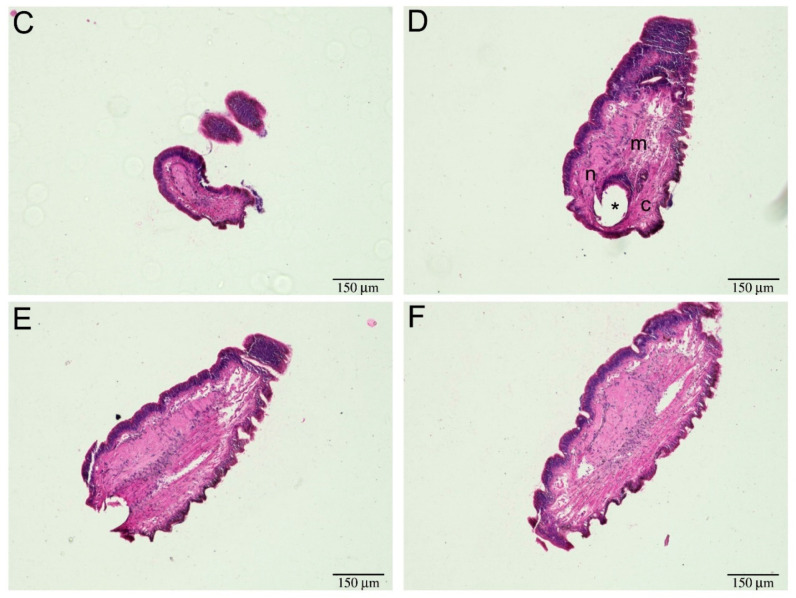
Base-to-tip histological analysis of *P. canaliculata* model cephalic tentacle. Longitudinal sections of tentacle tip. (**A**) Illustration summarizing cephalic tentacle components; the rectangle relates to the portion pictured in the histological micrographs (**B**) The first slice collected from tentacle tip extremity (t), is the only cross section presented in this panel. The neural component was still very clear. The position of the eye (e) and the oral tentacle (ot) has been reported for helping orientation (**C**–**F**) Longitudinal sections of the tip of the cephalic tentacle, moving from the dorsal to the ventral face, representing the whole tentacle thickness. Moving along the dorsal–ventral axis, the histological micrographs confirmed the presence of all the tissue components, but the tentacle nerve remained predominant. In (**D**,**E**) the hole in the tentacle (*) is an artifact due to the use of an entomology needle during fixation procedures. Abbreviations: see Figure 1.

**Figure 3:**
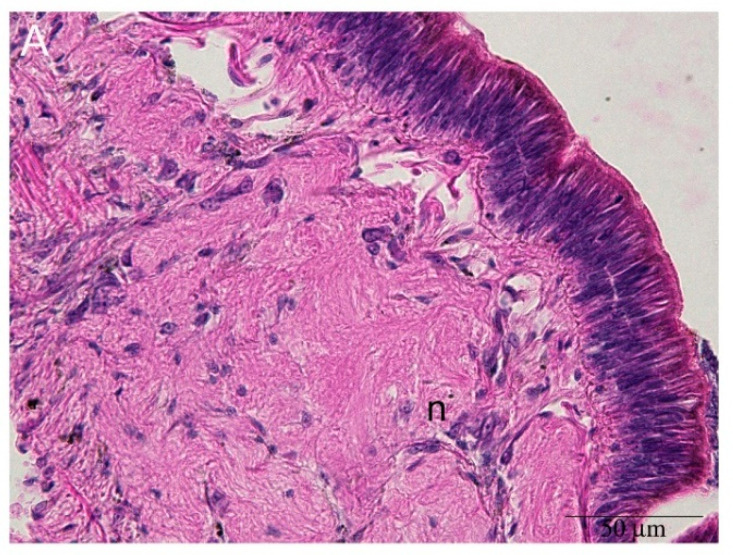

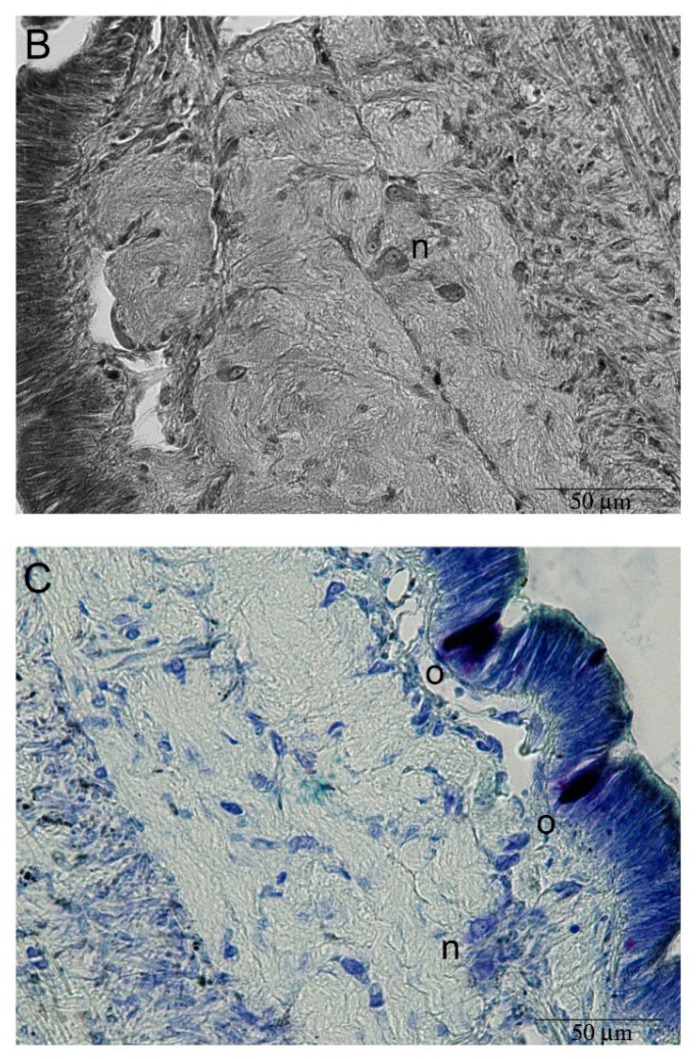
Neurons and olfactory cells in the cephalic tentacle of the apple snail *P. canaliculata*. (**A**) HE staining of tentacle nerve and epithelium. Neurons (n) were recognizable from their shape, while sensory cells are not recognizable among the epithelial cells; (**B**) Grayscale image derived from Masson’s trichrome staining. It was possible to identify a chain of neurons (n) along the tentacle nerve, but not olfactory cells; (**C**) Nissl staining: neurons (n) and olfactory cells (o) were recognizable, as a consequence of their shape and staining, respectively. Olfactory cells (o) displayed an intense blue-to-purple color, among the light blue epithelial cells [23,24].

Mayer’s hematoxylin/eosin (HE) and Masson’s trichrome staining, allowed the identification of the different tissues in *P. canaliculata* cephalic tentacle. A similar structural organization was found in all the tentacles, regardless of age and side of collection, therefore one entire left tentacle is reported to represent all the tentacles analyzed (Figure 1 and Figure 2). The tentacle presented the same histological organization throughout its length, as no significant histological differences were observed between the tentacle base and the tip (Figure 1 and Figure 2). The tentacle presented an external epithelium, and longitudinal muscles embedded in a loose connective tissue. The musculature was more represented along the eye-side of the tentacle while, along the oral side, a nerve was evident (Figure 1). This neural structure presented a conical shape from the base to the tip of the tentacle. At the base of the tentacle, the neural structure enlarged into a spherical shape, occupying the ventral part of the organ. (Figure 1E,F). As a consequence of the reduced muscle and connective tissues at the tentacle tip, the tentacle nerve became the predominant component (Figure 2). The nerve included neurons of different sizes (Figure 3B,C; Supplementary Figure S1).

**Figure 4:**
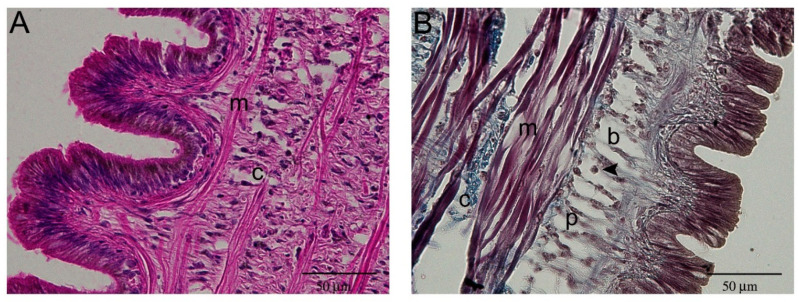
Blood-containing lacunae were located below the external epithelium on both sides of the tentacle. (**A**) HE staining displayed the alternate and regular pattern of muscle (m) and connective fibers (c); (**B**) Masson’s trichrome staining helped to identify the different cell types that could be observed in blood-containing lacunae (b). The cells (p) resembling pillar cells observed in fish gills [33] were stained like connective tissue (i.e., light blue). Hemocytes (arrowhead) could also be observed in the lacunae.

At higher magnification, the simple ciliated epithelium outlining the surface of the cephalic tentacle, contained cells positive for Nissl staining (Figure 3). Below the epithelium, blood-containing lacunae [23,24] were visible. Blood-containing lacunae were formed by a delicate net of connective fibers and by columnar cells connecting the basal membrane to the sub-epithelial connective tissue (Figure 3A and Figure 4). The lacunae of model tentacles contained a few hemocytes (Figure 4B), while no hemocytes were observed interspersed among the tentacle tissues (Figure 3A).

### 2.2. Tentacle Regeneration

**Figure 5:**
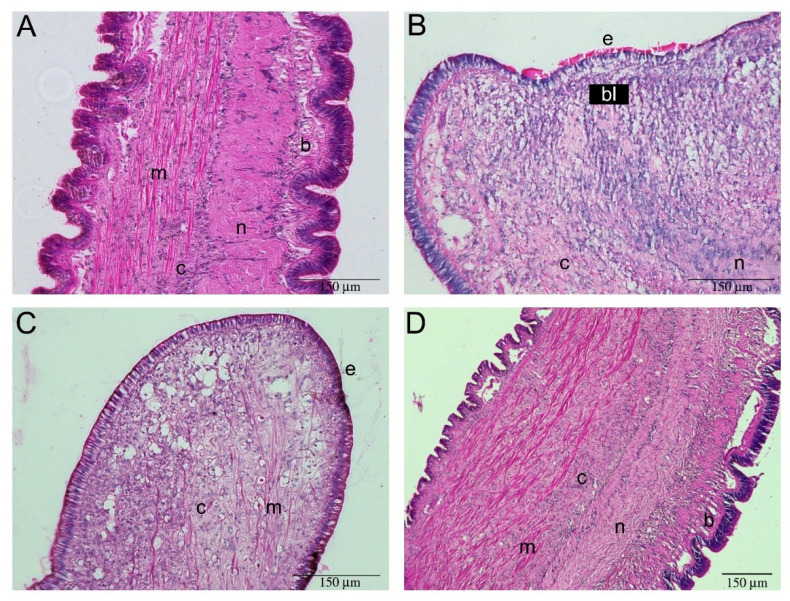
Blastema evolution during cephalic tentacle regeneration. (**A**) Model tentacle; (**B**) Regenerating tentacle at 48 hpa. Blastema (bl) was visible, as a mass of undifferentiated cells, under the newly formed epithelium (e). At this stage, the basal lamina under the new epithelium was not formed, yet. Below the blastema, the intact connective (c) and nervous (n) tissues were visible; (**C**) At 72 hpa, below the new epithelium (e), loose connective tissue and muscular fibers started to be evident, above the better organized connective tissue (c) and muscular (m) fibers; (**D**) Regenerated tentacle at 3 mpa. All tissue components were recognizable. Abbreviations: b, blood containing lacunae; c, connective tissue; m, muscle fibers; n, tentacle nerve.

**Figure 6:**
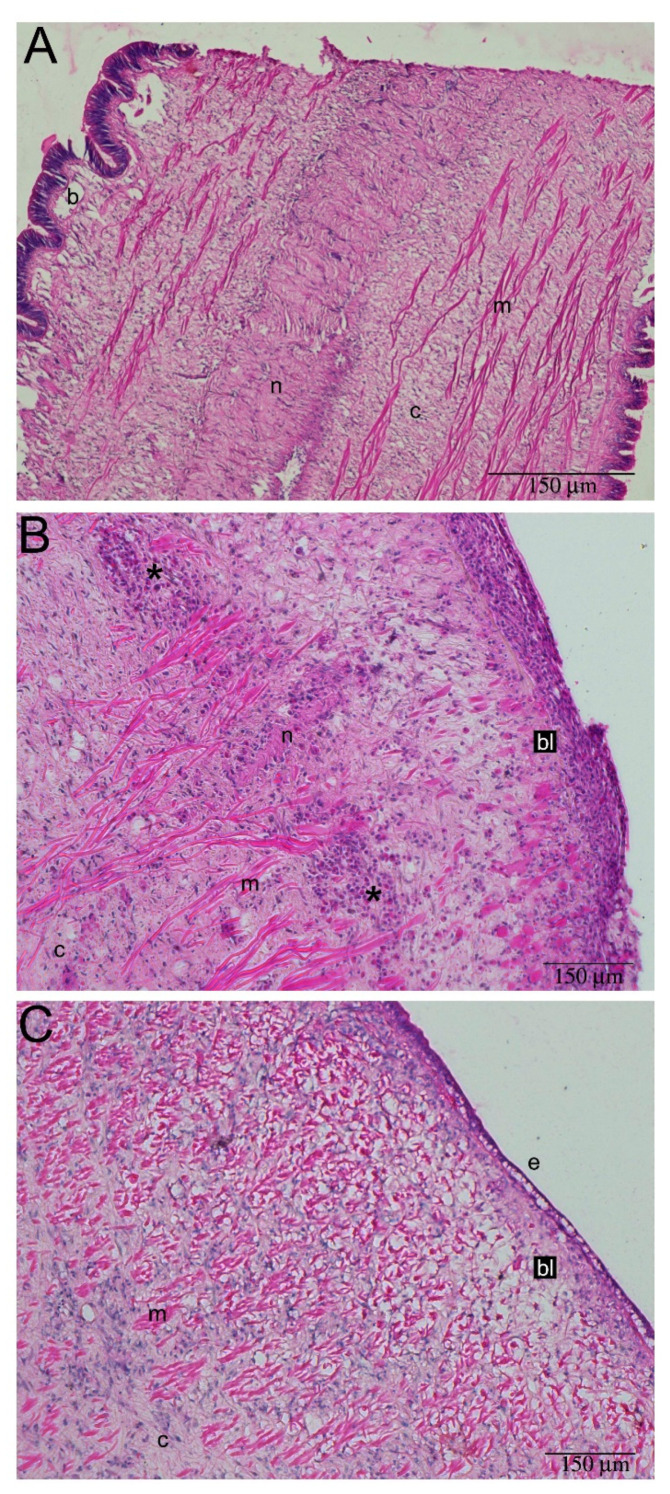
Hemocytes are present during early cephalic tentacle regeneration. (**A**) Cephalic tentacle immediately after amputation; (**B**) At 12 hpa, the wound site was recognizable due to the interruption in epithelial continuity. The thick blastema (bl) included several undifferentiated cells. Below the blastema, at the boundary between regular muscle (m) and nerve (n) fibers, large hemocyte aggregations (*) were observed; (**C**) At 24 hpa, the external epithelium (e) started to be recognizable and the blastema (bl) looked thinner. Hemocyte aggregates described at 12 hpa were not observed. Abbreviations: b, blood containing lacunae; c, connective tissue.

Adult *P. canaliculata* could regenerate cephalic tentacles within 3 months post-amputation (mpa) (Figure 5D; Figure S2), but the regenerating process started immediately after wounding (Figure 5 and Figure 6). At 48 h post-amputation (hpa), the wound was completely closed, and the animals showed a new-formed, thin and pigmented external epithelium at the amputation site, while the basal membrane was not evident, yet. Below the reconstructed epithelium, an active blastema was visible; tissue differentiation was not clear at this stage. Differentiated cells and tissues, such as neurons and muscles could be found in proximity of the wound area, just below the blastema (Figure 5B). In 72 hpa samples, the regenerating tentacle showed better differentiated and new-formed tissues (Figure 5C), that differed from the blastema described for the 48 hpa time-point. At 72 hpa, the main structural components were recognizable, and further observations at 96 and 120 hpa confirmed the tentacle growth without further tissue rearrangements (data not shown). At 3 mpa, the regenerated tentacle was indistinguishable from the controls. No differences were observed in tentacle histology, as nerves, muscles, connective tissue, blood-containing lacunae and sensory cells could be recognized (Figure 5A,D).

### 2.3. Hemocyte Identification during Early Blastema Formation

**Figure 7:**
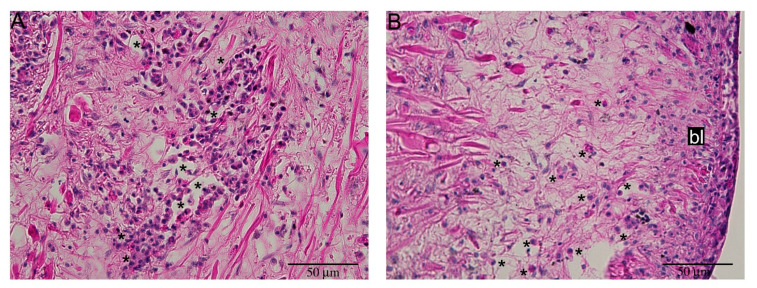
Detail of hemocyte distribution during early regeneration. (**A**) Intact tissue adjacent to undifferentiated blastema showed extended clumps of hemocytes (*); (**B**) Isolated hemocytes (*) infiltrated blastema (bl) base, distributing into the undifferentiated tissue.

Hemocytes were not evident in the regenerating tentacle at 48 and 72 hpa (Figure 5). At 12 and 24 hpa, tentacle blastema (i.e., a mass of undifferentiated cells) was visible. Group I and II hemocytes infiltrated the blastema at 12 hpa (Figure 6 and Figure 7). Beside the hemocytes dispersed into the blastema, other hemocytes were present in blood-containing lacunae, and in the intact tissues surrounding the active blastema (Figure 7). At the 24 hpa time-point, epithelial cells were recognizable at the tip of the wound site and blastema extension started to reduce. Hemocyte presence was less marked (Figure 6C).

### 2.4. Automated Hemocyte Count in Control and 12 hpa Slides

**Figure 8:**
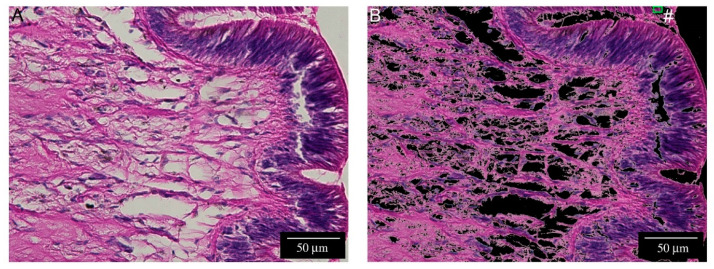

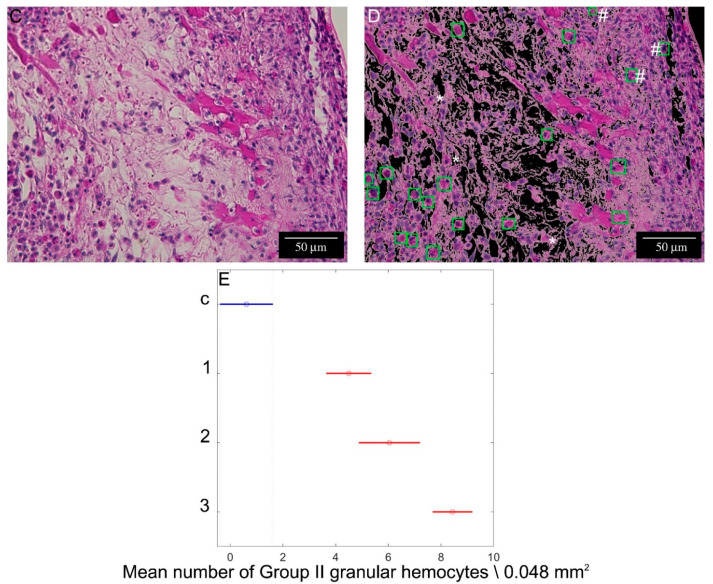
Schematic representation of the MatLab^®^-based approach for computer-assisted hemocyte identification. Color thresholds and shape filters were applied to remove unfit colors and shapes, and to automatically single-out objects corresponding to Group II granular hemocytes (green boxes). The program was trained to recognize Group II granular hemocytes in images from control (**A**) and amputated snails (**C**). The results of automatic identification of hemocytes (green squares) were shown in (**B**,**D**), respectively. Misclassification (false positive (#) and false negative (*) hits) was infrequent and observed in specific and recognizable situations, such as the border of the blood-containing lacunae, and rare muscle fibers sectioned along unusual angles. (**E**) Computer-assisted hemocyte count in the control (c, blue) and regenerating tentacles 12 hpa (1, 2, 3, red). Mean hemocyte count ± S.D, from ANOVA, is represented. The surface of 0.048 mm^2^ corresponds to the microscopic field pictured with a EVOS M5000 Imaging System (ThermoFisher Scientific, Milan, Italy) using a plan S-APO CC 40×/0.95 objective. Non-overlapping segments represent significantly different data according to the Tukey–Kramer test (*p* < 0.05).

After the application of color thresholds and shape filters, Group II granular hemocytes have been correctly classified, with negligible misclassifications. Automated hemocyte count revealed a significant difference between control and 12 hpa snails (Figure 8). The mean number of Group II granular hemocytes registered for regenerating tentacles was from 5 to 9 times higher than in controls.

## 3. Discussion

The applications of regenerative medicine [34] and the evolutionary conservation of regeneration processes [35] collected the interests of scientists of different backgrounds, prompting experiments in evolutionary distant models [2]. The role of the immune system in the regeneration has been analyzed from different perspectives, with evidence suggesting either a promoting or an inhibiting role [8,10]. Molecular information about *P. canaliculata* and its hemocytes is increasing [31,36,37,38] but, to our knowledge, no specific cell markers have been described for tracking circulating [25,31] or tissue-resident [30,36,39] hemocytes. In absence of cell markers, the most advanced techniques of image analysis [28] cannot be applied, and other methods must be developed to time-effectively and reliably document hemocyte presence in *P. canaliculata* tissues. This could also pave the way for studies concerning the effects of hemocyte depletion on organ regeneration. In most cases, the quantification of tissue-resident hemocytes is performed cell by cell at the microscope, by a trained operator [30,39,40]. The recognition and counting of hemocytes circulating into the hemolymph can be effectively performed by flow cytometry-based protocols [25,29,30], whereas counting of tissue-resident hemocytes is a time-consuming approach. Hence, histological protocols, optical microscopy and a novel computer-assisted image analysis were here combined to document the presence of the hemocytes in the regenerating tentacles.

The presence of hemocytes has been detected only in the early blastema, i.e., at 12 hpa. The computer-assisted counting process can automatically identify and count Group II granular hemocytes from bright field microscopy images, with a minimal possibility of misinterpretation. The future identification of further morphological markers and/or an improved version of our plug-in, may help to extend our image analysis protocol also to Group I and agranular Group II hemocytes.

Consistently with histochemical observations, the automated image analysis allowed us to detect a significant increase in granular hemocytes in close proximity to the blastema at 12 hpa. The rapid decrease in hemocytes observed at the microscope in 24, 48 and 72 hpa samples, suggests that the role of *P. canaliculata* hemocytes could be relevant in the early onset of the regeneration process, as observed in other gastropods. During the wound repair of the land snail *Limax maximus*, the hemocytes quickly accumulate in the proximity of the wound to engulf cellular debris. *L. maximus* hemocytes express cytokine-like molecules and the effects of heterologous growth factors suggest that the hemocyte role could also include the active promotion of wound repair and tissue regeneration [11]. In the freshwater snail, *Physella heterostropha*, the role of unknown hemocyte-derived growth factors is indispensable for allowing in vitro regrowth of severed neurites from cultured neurons [41]. Similar observations have been reported in the sea slug *Aplysia californica* [12]. Beside neural components, molluscan hemocytes also cooperate to the regeneration of other components such as the shell [42,43]. Accordingly, the proteomic analysis of *P. canaliculata* circulating hemocytes evidenced numerous proteins related to the immune response and cell signaling, confirming the potentialities of hemocytes to directly influence the function and metabolism of other cells [31].

The active intervention of the nerve-associated hemocytes in the regeneration of neural components has been well-documented in the annelids *Hirudo medicinalis* and *Dendrobaena veneta* [9,10]. In the leech *H. medicinalis*, the neural cells synthesize immune-related factors that seem fundamental for microglial recruitment and the neural cord regeneration [44,45]. On the basis of the existing literature [9,10,12,31,41,42,43,44,45,46,47,48], it is tempting to speculate that the role of hemocytes in regenerating cephalic tentacle of *P. canaliculata* is not only that of scavenger cells removing the cell debris immediately after the amputation, but also to play an active role in the regrowth of neural and other tissue components. Further studies are necessary for gaining an in-depth comprehension of the roles played by hemocytes in the adult regeneration of *P. canaliculata*. In these respects, the first protocol developed for counting tissue-associated hemocytes without the need of specific cell markers may represent a helpful tool for studying the potential involvement of immune-related cells in the adult regeneration of sensory organs.

## 4. Materials and Methods

### 4.1. Animal Maintenance

*Pomacea canaliculata* were reared and bred in aquaria containing constantly aerated tap water at 25 ± 1 °C, no recirculation system and a 14:10 h light–dark photoperiod, with a maximal density of 1 animal/2 L of water. Twice a week, tanks were deeply cleaned and approximately 80% of the water was changed. Immediately after water change, animals were fed with mixed types of green salad, and were allowed to feed at libitum until the next water change.

Adult (6- to 10-month-old) snails were used for regeneration experiments. Three snails were used for each time-point in regeneration experiments.

For the histological description of model tentacles, both the left and right cephalic tentacles of 6 animals were studied. The age range of the snails was from 3 months up to 1-year old. No differences were observed in tentacles from animals of different age (data not shown).

### 4.2. Tentacle Amputation and Processing

The animals were reversibly anaesthetized by placing them in ice for 20 min, and the head and tentacles were then accessible by lowering the operculum. Tentacle amputations were quickly conducted under the stereo microscope, with spring scissors (F.S.T^®^ 15006-09 Besozzo, Italy). For documenting tentacle regeneration, we amputated only the cephalic tentacle, at 3 ± 1 mm from its base, paying attention not to damage the eye and surrounding tissues. For histological analysis of model tentacles, the cut was performed laterally across the base of the eye peduncle and tentacle origin, to keep the eye together with the tentacle (Figure S3). All the collected samples where fixed in freshly made Bouin’s solution (15 parts of picric acid saturated water solution (Sigma-Aldrich, Merck Life Sciences SrL, Milan, Italy), 5 parts of formaldehyde 40% (Sigma-Aldrich, Milan, Italy), 1 part of acetic acid (Carlo Erba, Milan, Italy)) for 6–8 h at room temperature (RT). After fixation, the model tentacles were further cut into 3 portions of 18 ± 2 mm each: base (with the eye), middle part, and tip of the tentacle. The samples were then conserved in 70% ethanol at −20 °C before dehydration and inclusion procedures.

### 4.3. Post-Surgery Maintenance and Blastema Collection

After surgery, the animals were kept into plastic net cages (25 × 20 × 40 cm^3^; max 3 animals per cage), placed into 120 L tanks. Different time-points were fixed for the collection of regenerating tentacles: 12, 24, 48 and 72 hpa. Regenerating tentacles were also collected 4, 5, 6 and 7 days post amputation (data not shown). The final time-point was set at 3 mpa. This time-point probably exceeds the minimal time required for the full regeneration of the cephalic tentacles but, in the absence of information about the gene expression recovery, we fixed the last time-point after all the regenerated tentacles were visually indistinguishable from the original ones, both in terms of length, width and color (Figure S2). The first time-point was fixed at 12 hpa to allow for snail recovery after the first surgery. Amputation of regenerating tentacles was made as described above. No snails died during the experiments.

### 4.4. Histology

Specimens were dehydrated with ethanol (70° absolute 100°), cleared (90 min) in xylene (Carlo Erba, Milan, Italy), before paraffin wax infiltration. Paraffin wax infiltration consisted of two steps of 3 h each, followed by wax block preparation. Samples were transferred in new batches of melted paraffin at each step, in order to remove xylene. During the embedding, the specimens were oriented by using tentacle pigmentation and, where available, the annexed eye. Paraffin-embedded tentacles were sectioned into 7 µm thick slices from dorsal to ventral face. Seriate slices where distributed onto lots of 4 slides, for a total of 5 slices per slide. Mayer’s Hematoxylin and Eosin (HE), Nissl and Masson’s trichrome staining (Bio-Optica, Milan, Italy) were performed on slide 1 to 3, respectively, while slide 4 was kept as a backup. HE and Nissl staining were performed following the standard protocols [11], while Masson’s trichrome was performed accordingly to the kit’s instructions. Unless otherwise stated, all the reagents were purchased from Sigma-Aldrich (Merck Life Sciences, Milan, Italy)

### 4.5. Image Acquisition and Analysis

Images were taken from 12 hpa animals and investigated. Control images were taken from a 0 hpa animal, assuming that regeneration processes have not started yet. The images were acquired using EVOS M5000 Imaging System (ThermoFisher Scientific, Milan, Itay) with a plan S-APO CC 40×/0.95 objective, that offered the best combination between microscopic field size and cell resolution. In view of the computer-assisted hemocyte count on 12 hpa samples, alternate slices were chosen from all the available HE-stained slides. Image capture settings (i.e., light intensity, exposure and gain) were initially set on controls, in order to guarantee a comparable background for all the images under analysis.

### 4.6. Computer-Assisted Image Analysis

Computer-assisted Group II granular hemocyte count was performed by applying an image color threshold, multivariate image analysis (MIA) and object identification. A plug-in was implemented by some of the authors in the MATLAB^®^ R2020a (Mathworks, MA, USA) environment; this plug-in uses also routines from the Image Processing Toolbox™ Release 2020a (Mathworks, MA, USA).

In more detail, the automated procedure to count hemocytes was developed as follows:

- Assembling a training image to model hemocytes.

Ten images containing clear representations of Group II hemocytes were selected for assembling a training image. From each of the 10 images used to train the algorithm, the background was removed by fixing a threshold level for the green and blue color channels (Supplementary Figure S4).

The hemocytes were manually singled out on each of the 10 images and the pixels corresponding to an area including only the identified hemocytes were mounted on a new image, i.e., the training image, constituted only by hemocytes (Supplementary Figure S4). It is worth noting that this step, consisting of manually counting the hemocytes is required only once during the calibration phase, to assemble a representative reference training image on which the model is built. Afterwards, any image can be projected on this model without requiring any manual assessment.

- Applying MIA on the training image.

MIA captures color and spatial information in multi-channel images [49]. It consists of unfolding each color channel pixelwise, obtaining a matrix with as many rows as pixels (nx*ny) and as many columns as color channels (RGB in our case). The column number is then increased by adding the color intensities of the neighboring pixels in a window of a given size (in our case, a window of 2 pixels in any directions). This matrix is then subjected to PCA, and the score vectors obtained are refolded in “score images” that show the main spatial and color features. In our case, the hemocyte salient features are clearly recognizable on the fourth principal component (PC4) (Supplementary Figure S4). A threshold value was set on PC4 to isolate the pixel corresponding to the hemocytes (Supplementary Figure S4). Multiple PCs can be utilized to model any information. This, however, was unnecessary in our case.

- Computer-assisted hemocyte count

Sixty images from the control and 82, 46 and 111 images, from the three 12 hpa snails have been processed. Each image under analysis underwent a MIA procedure after applying a green-blue level threshold to remove the background, as described above. After this first processing, tested images were analyzed through PCA. By applying the PC4 threshold value, the projected score image is obtained. On this image, hemocyte identification is applied by standard methods [50,51] and shape parameters are calculated, i.e., area, width, height, circularity and solidity, which are used to exclude the objects presenting a color/texture similar to Group II granular hemocytes but unfit shapes or sizes. The objects remaining after exclusion steps were localized in the raw images and automatically counted as hemocytes (Supplementary Figure S4). The number of false positives and false negatives was visually checked.

In the present article, granular Group II hemocytes (HE stained) were the only hemocytes counted with the developed automatic plug-in. The application of the same approach to Group I and agranular Group II required manual output corrections by the operator. This was because in some cases, section plane and tissue staining made other tissue components too similar to Group I and Group II agranular hemocytes. As a consequence of these negligible shape and color differences, the levels of false positivity required a time-consuming manual elimination of false-positive identifications. New plug-ins are currently under development, by using wavelet filter to fine tuning of small differences in color-textural patterns [52], in order to automatically count also the hemocytes excluded from this analysis. Conversely, due to the specific staining and color distribution, granular Group II hemocytes were identified with high precision and accuracy (Supplementary Table S1). In order to assess if there was a significant difference among the number of granular Group II hemocytes between the control and 12 hpa animals, one factor analysis of variance (ANOVA) has been applied on the counting and the Tukey–Kramer test [53] has been used for assessing the significance of pair means differences.

## Supplementary Materials

The following are available online at https://www.mdpi.com/article/10.3390/ijms22095023/s1, Figure S1: Neurons after Nissl staining in the cephalic tentacle of the apple snail *P. canaliculata*. Neurons of different sizes (small, asterisks; large, arrowheads) were observed. Figure S2: Comparison between uncut (**A**,**C**) and fully regenerated (**B**,**D**) tentacles. Regenerated tentacles were indistinguishable from controls, both in length and width. White arrowhead indicates the cephalic tentacle origin and eye, Figure S3: Illustrations of the cut sites (red dotted line) for (**A**) regeneration experiments and (**B**) model tentacle histological analysis, Figure S4: Methodological framework of computer assisted hemocyte count. (**A**) Ten images were chosen, where the Group II granular hemocytes were observed most clearly. (**B**) Green and blue color channel thresholds were applied on the original images to remove the background. (**C**) Hemocytes were manually extracted from (**B**) and fused into a singular training image. (**D**) The PC score image that modelled the hemocytes most clearly was chosen (other PCs not shown). (**E**) A threshold was applied to the score values, to exclude all the pixels that did not conform to the hemocyte coloring. (**F**) Object size and shape identification was applied to remove all the objects that did not conform to the standard hemocyte morphology, which is circular. (**G**) Example of one image under analysis. (**H**) The identical color thresholds applied for (**B**) were applied to the green and blue color channels. (**I**) Object identification was performed, according to the model depicted in (**A**–**F**), Table S1: Comparison between manual and computer-assisted Group II granular hemocyte cell count. Group II hemocytes were identified with high precision and accuracy in 20 randomly chosen microscopic fields of control and 12 hpa amputated snails.
