# Supplementary material for: A New Protocol of Computer-Assisted Image Analysis Highlights the Presence of Hemocytes in the Regenerating Cephalic Tentacles of Adult Pomacea canaliculata
Source: Int J Mol Sci. 2021 May 9;22(9):5023. doi: 10.3390/ijms22095023 (PMC8126035; doi:10.3390/ijms22095023)
Supplement: Supplementary file 1 [file ijms-22-05023-s001.zip › ijms-1157896-supplementary.pdf]

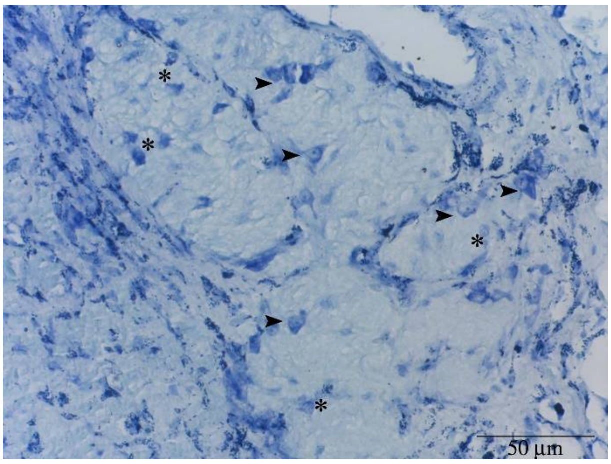

Figure S1. Neurons after Nissl staining in the cephalic tentacle of the apple snail *P. canaliculata*. Neurons of different sizes (small, asterisks; large, arrowheads) were observed.

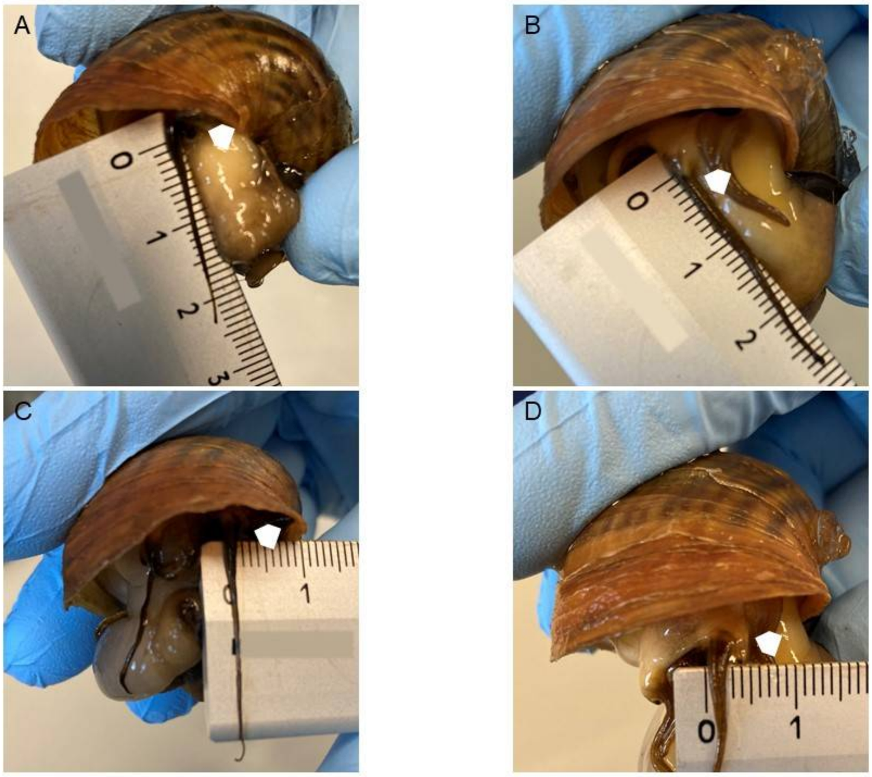

Figure S2. Comparison between uncut (**A**,**C**) and fully regenerated (**B**,**D**) tentacles. Regenerated tentacles were indistinguishable from controls, both in length and width. White arrowhead indicates the cephalic tentacle origin and eye.

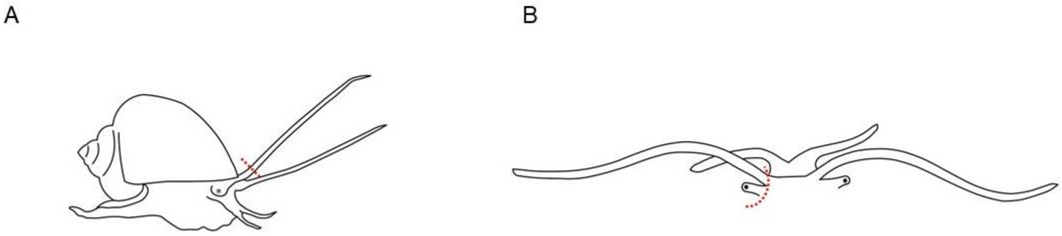

Figure S3. Illustrations of the cut sites (red dotted line) for (**A**) regeneration experiments and (**B**) model tentacle histological analysis.

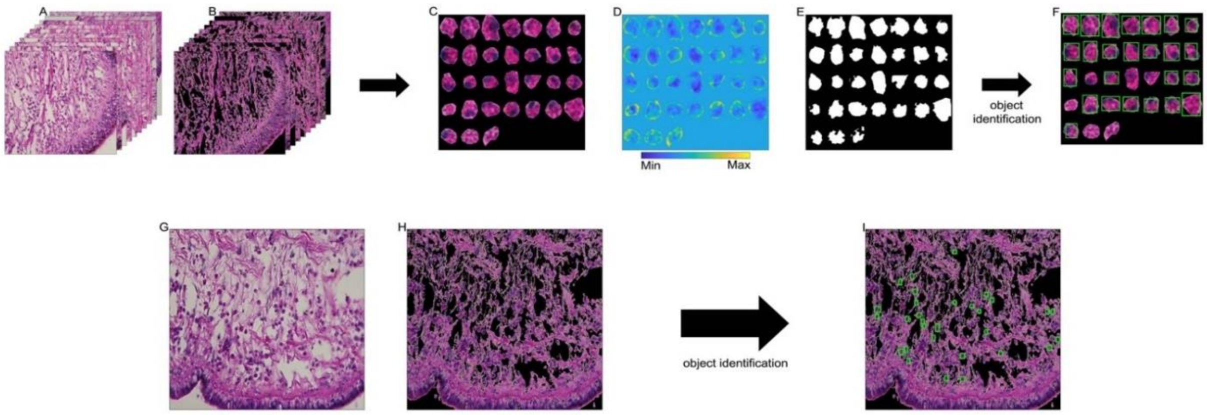

Figure S4. Methodological framework of computer assisted hemocyte count. (**A**) Ten images were chosen, where the Group II granular hemocytes were observed most clearly; (**B**) Green and blue color channel thresholds were applied on the original images to remove the background; (**C**) Hemocytes were manually extracted from (**B**) and fused into a singular training image; (**D**) The PC score image that modelled the hemocytes most clearly was chosen (other PCs not shown); (**E**) A threshold was applied to the score values, to exclude all the pixels that did not conform to the hemocyte coloring; (**F**) Object size and shape identification was applied to remove all the objects that did not conform to the standard hemocyte morphology, which is circular; (**G**) Example of one image under analysis; (**H**) The identical color thresholds applied for (**B**) were applied to the green and blue color channels; (**I**) Object identification was performed, according to the model depicted in (**A**–**F**).

Table S1. Comparison between manual and computer-assisted Group II granular hemocyte cell count. Group II hemocytes were identified with high precision and accuracy in 20 randomly chosen microscopic fields of control and 12 hpa amputated snails.

Specimen	Manual Count	Computer-Assisted Count
Control #1	0	0
Control #2	0	1
Control #3	0	0
Control #4	0	0
Control #5	0	0
12hpa #1	5	9
12hpa #2	18	19
12hpa #3	19	21
12hpa #4	10	12
12hpa #5	12	14
12hpa #6	38	34
12hpa #7	8	5
12hpa #8	25	30
12hpa #9	12	12
12hpa #10	26	26
12hpa #11	6	4
12hpa #12	21	22
12hpa #13	5	5
12hpa #14	10	11
12hpa #15	22	20
